# Neurosonographic Classification in Premature Infants Receiving Omega-3 Supplementation Using Convolutional Neural Networks

**DOI:** 10.3390/diagnostics14131342

**Published:** 2024-06-25

**Authors:** Suzana Zivojinovic, Suzana Petrovic Savic, Tijana Prodanovic, Nikola Prodanovic, Aleksandra Simovic, Goran Devedzic, Dragana Savic

**Affiliations:** 1 Department of Pediatrics, Faculty of Medical Sciences, University of Kragujevac, Svetozara Markovica 69, 34000 Kragujevac, Serbia; zivojinovicsuzana@yahoo.com (S.Z.); tijanaprodanovic86@gmail.com (T.P.); aleksandra.simovic@yahoo.com (A.S.); drsavicdragana@gmail.com (D.S.); 2 Center for Neonatology, Pediatric Clinic, University Clinical Center Kragujevac, Zmaj Jovina 30, 34000 Kragujevac, Serbia; 3 Department for Production Engineering, Faculty of Engineering, University of Kragujevac, Sestre Janjic 6, 34000 Kragujevac, Serbia; petrovic.suzana@gmail.com (S.P.S.); devedzic@kg.ac.rs (G.D.); 4 Department of Surgery, Faculty of Medical Sciences, University of Kragujevac, Svetozara Markovica 69, 34000 Kragujevac, Serbia; 5 Clinic for Orthopaedic and Trauma Surgery, University Clinical Center Kragujevac, Zmaj Jovina 30, 34000 Kragujevac, Serbia

**Keywords:** hypoxic-ischemic encephalopathy, ultrasonography, density difference, convolutional neural network, neonates, intensive care, image classification, brain parenchyma, choroid plexus, medical imaging

## Abstract

This study focuses on developing a model for the precise determination of ultrasound image density and classification using convolutional neural networks (CNNs) for rapid, timely, and accurate identification of hypoxic-ischemic encephalopathy (HIE). Image density is measured by comparing two regions of interest on ultrasound images of the choroid plexus and brain parenchyma using the Delta E CIE76 value. These regions are then combined and serve as input to the CNN model for classification. The classification results of images into three groups (Normal, Moderate, and Intensive) demonstrate high model efficiency, with an overall accuracy of 88.56%, precision of 90% for Normal, 85% for Moderate, and 88% for Intensive. The overall F-measure is 88.40%, indicating a successful combination of accuracy and completeness in classification. This study is significant as it enables rapid and accurate identification of hypoxic-ischemic encephalopathy in newborns, which is crucial for the timely implementation of appropriate therapeutic measures and improving long-term outcomes for these patients. The application of such advanced techniques allows medical personnel to manage treatment more efficiently, reducing the risk of complications and improving the quality of care for newborns with HIE.

## 1. Introduction

Due to its immaturity, the brain of preterm infants is at risk for numerous lesions. The most common of these include intraventricular hemorrhage from the germinal matrix (IVH), ventricular system enlargement, cerebellar hemorrhage, and white matter damage known as periventricular leukomalacia (PVL) [1]. Brain injury resulting from perinatal asphyxia is known as hypoxic-ischemic encephalopathy (HIE). The incidence of HIE ranges from 1 to 7 cases per 1000 live births, with significantly higher numbers in developing countries [2].

Hypoxic-ischemic insult progresses through three phases: the primary phase, involving cell necrosis due to oxygen deprivation; the latent phase, characterized by reperfusion and temporary restoration of blood flow, which can lead to additional damage; and the secondary phase, involving apoptosis, or programmed cell death, which further aggravates brain injury in the days following the initial event. Based on clinical evaluation, which includes assessing the level of consciousness, muscle tone, tendon reflexes, autonomic disturbances, and the presence of seizures along with EEG findings, HIE can be graded as mild, moderate, or severe using the Sarnat score [3,4].

Neuroradiological methods that enable visualization of brain structures include ultrasonography (US), computed tomography (CT), and magnetic resonance imaging (MRI). Ultrasound of the central nervous system (CNS US) is the most commonly used diagnostic method for studying brain lesions and their potential outcomes. Due to its accessibility, safety, reliability, and low cost, ultrasound has become an integral part of equipment in almost all intensive care units [5].

Brain ultrasonography is performed through the anterior fontanelle, which remains open and serves as an acoustic window until the tenth month of life. Convex probes with frequencies of 5–6.5 MHz or 5–10 MHz are used for this procedure, and they must be small enough to allow visualization of brain structures through a very narrow fontanelle. Visualization of superficial brain structures can be achieved using linear probes. Standard ultrasound planes include coronal and parasagittal sections. The coronal section provides an overview of the brain hemispheres and allows their comparison, while the parasagittal section enables visualization of the ventricular and paraventricular structures [6,7].

Diagnosis of PVL based on brain ultrasound is performed by comparing the echogenicity of the white matter (brain parenchyma) and the choroid plexus. The echogenicity of the white matter can be classified into three categories [8]:

1. Normal echogenicity: the echogenicity of the white matter is normal compared to the choroid plexus, usually without cystic changes.
2. Mild echogenicity: mild hyperechogenicity of the white matter, which may indicate the early stages of PVL but without the formation of cystic changes.
3. Intensive echogenicity: significant hyperechogenicity of the white matter, which may indicate more severe lesions and could lead to cystic changes.

Based on CNS ultrasound, PVL is described through four grades [8]:

Grade I: Transient hyperechogenicity of the white matter without the formation of cystic changes (may correspond to normal or mild echogenicity);
Grade II: Presence of cystic changes in the frontal or frontal-parietal regions (may correspond to mild or intensive echogenicity);
Grade III: Cystic changes affecting the parieto-occipital regions (may correspond to intensive echogenicity);
Grade IV: Cysts extending to the subcortical region, resembling porencephalic cysts (may correspond to intensive echogenicity).

The drawbacks of this classification in daily practice include the subjectivity in assessing the degree of hyperechogenicity, which can lead to diagnostic variations among different examiners. Additionally, in extremely preterm infants, especially those younger than 28 weeks of gestation, the choroid plexus may be more echogenic, increasing the risk of cystic changes occurring without previously observed hyperechogenicity. These factors can complicate the accurate diagnosis of PVL and require additional attention and experience in interpreting ultrasound findings [9].

In contrast to cystic PVL, for the diagnosis of diffuse white matter injuries, such as diffuse white matter lesions (DWMLs), MRI is considered a more reliable method. MRI allows for more detailed visualization of brain tissue and detects even the smallest changes in the structure and composition of white matter, making it very useful for diagnosing diffuse white matter injuries [10].

Therapeutic hypothermia is applied as the only effective treatment for newborns with moderate to severe HIE. This therapy involves controlled lowering of the infant’s body temperature to approximately 33–34 °C for a specific period, usually 72 h, to reduce brain damage caused by oxygen deprivation during birth. However, despite the application of therapeutic hypothermia, a significant number of newborns still develop serious neurological impairments [2,5].

Long-chain omega-3 polyunsaturated fatty acids (PUFAs), particularly docosahexaenoic acid (DHA), have been shown to be beneficial in therapy following acute brain injuries due to their neuroprotective, anti-inflammatory, and antioxidant properties. DHA is an essential fatty acid that is a key component of the structure of cell membranes in the brain and central nervous system. Some studies have suggested that DHA supplementation may have a positive therapeutic effect following acute brain injuries. DHA may help reduce inflammation and oxidative stress, which can contribute to minimizing further brain tissue damage and accelerating the recovery process [11].

The introduction of artificial intelligence (AI) in medicine is significantly transforming various areas of healthcare, and neonatology is no exception. Artificial intelligence, particularly convolutional neural networks (CNNs), is bringing revolutionary changes to the diagnosis and treatment of newborns. A CNN, as a part of deep learning, enables efficient analysis of medical images by recognizing complex patterns and anomalies. There are several reasons why it is crucial to use CNNs in neonatology. First, rapid and accurate diagnosis is essential for timely intervention and treatment. Newborns are especially vulnerable, and every moment is critical to preventing permanent damage. Second, a CNN reduces the risk of human error, which is particularly important in the stressful and demanding situations of neonatal units. Third, the use of CNNs allows neonatologists to focus on critical clinical observations and decision-making while algorithms take over the routine tasks of image analysis [12,13].

One example of the application of a CNN in neonatology is the identification of catheters and tubes on radiological images. R.D.E. Henderson and colleagues developed a CNN-based tool using ResNet-50, which has demonstrated significant utility in medical diagnostics [12]. Additionally, A. O’Shea et al. created a CNN for the detection of neonatal seizures, a complex and precise task in interpreting EEG waves, providing objective support in identifying seizures and timely alerting clinicians [13].

Furthermore, S. Ervural and M. Ceylan used a CNN to assess temperature and thermal symmetry, which is crucial for monitoring health status and predicting potential risks in newborns [14]. Additionally, S.F. Abbasi et al. demonstrated that the identification of quiet sleep in infants using CNNs provides a fast, inexpensive, and reliable method for monitoring sleep, which is important for assessing brain maturation and identifying potential health issues [15].

In the field of ophthalmology, N. Salih et al. developed a CNN-based classification algorithm for the early detection and treatment of retinopathy, reducing the risk of childhood blindness. These advanced algorithms enable faster and more accurate diagnostics, reducing the burden on physicians and minimizing the risk of human error [16].

The integration of CNNs into the daily practice of neonatology improves treatment outcomes, reduces mortality rates, and enhances the quality of care provided to the youngest patients. These advanced algorithms enable faster and more accurate diagnostics, reducing the burden on physicians and minimizing the risk of human error.

Given the current importance of the topic and the intensive research in the field of HIE, as well as the potential improvements in existing diagnostic procedures, the focus of this research is on developing a methodology for assessing the difference in density of brain ultrasound images, as well as classifying these images using a CNN. This approach aims to enhance understanding and achieve objectivity in assessing brain damage in newborns with HIE.

## 2. Materials and Methods

### 2.1. Patients

This study included 51 preterm infants born before 37 weeks of gestation who were hospitalized from the central and southwestern regions of Serbia (from 13 maternity hospitals) to the Center for Neonatology, Clinic for Pediatrics, University Clinical Center Kragujevac. Their gestational maturity ranged from 27 to 36 weeks. The type of delivery for each newborn was recorded, with 58.82% delivered by normal delivery and 41.18% by cesarean section. Birth weight ranged from 960 g to 3100 g. Asphyxia was established after analyzing gas exchange in arterialized capillary blood, accompanied by a progressive decrease in pO_2_, an increase in pCO_2_, and a decrease in blood pH levels.

The parents of the preterm infants were informed of the examination procedure in accordance with the rules of the Declaration of Helsinki and Good Clinical Practice, with the approval of the local ethics committee (approval number 01/22/26 from 24 January 2022), and they voluntarily agreed to participate in this study.

The primary criteria for including patients in this study were as follows:

- Preterm infants (born before 37 weeks of gestation) with signs of hypoxic-ischemic lesions of the CNS;
- Preterm infants who tolerated enteral feeding, allowing for supplementation from the 8th day of life;
- Preterm infants whose parents signed informed consent for participation in this study.

The exclusion criteria for this study were as follows:

- Preterm infants with congenital anomalies;
- Preterm infants whose supplementation was discontinued after the 8th day of life due to complete cessation of oral intake (for medical reasons);
- Preterm infants whose supplementation was discontinued after discharge but before the third month of life;
- Preterm infants whose parents did not sign informed consent for participation in this study.

All patients included in this study were given a daily supplement of 100 mg of omega-3 fatty acids starting from the eighth day of life.

For all cases included in this study, the following parameters were monitored:

- Maternal history: age, parity, gravidity, previous miscarriages, stillbirths, neonatal deaths, as well as acute and/or chronic illnesses;
- Anthropometric measurements of the newborn at birth: body weight, body length, head circumference, chest circumference, Apgar scores;
- Laboratory analyses: complete blood count, C-reactive protein, glucose levels, gas analysis (pH-CO_2_-O_2_-HCO_3_-BE) in arterialized capillary blood;
- Ultrasound scans: CNS brain images were monitored.

### 2.2. Statistical Analysis

To conduct a detailed analysis of the results for newborns who experienced brain asphyxia, descriptive statistics were applied to provide insight into key parameters. This analysis serves as the basis for further interpretation and discussion of the results.

Additionally, a comparative analysis of gas levels was performed within the first 6 h after birth. Another analysis was conducted two weeks after the introduction of omega-3 fatty acid supplementation. This was done once the respiratory function was stabilized and oxygen therapy was discontinued. A paired samples *t*-test was used for this comparison. This statistical approach allows for the identification of significant changes in gas analyses following asphyxiated lesions and after the respiratory stabilization of the patients.

### 2.3. Ultrasound Image Processing

**Figure 1:**
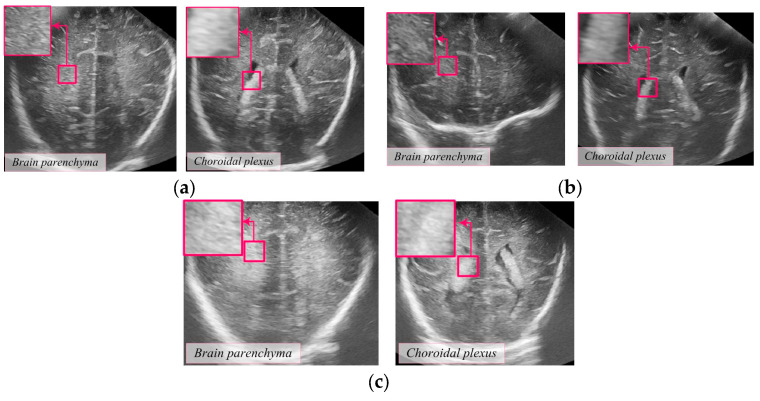
Ultrasound images of brain parenchyma and choroid plexus (magnification: 3×): (**a**) Normal, (**b**) Moderate, and (**c**) Intensive.

Previous studies on HIE in the brain mainly relied on the subjective assessment of physicians based on the density of brain ultrasound images of newborns [17,18,19]. The assessment was done by comparing two ultrasound images: the choroid plexus and the brain parenchyma. Based on these images, conclusions were made about whether the damage was Normal, Moderate, or Intensive. A Normal condition is characterized by homogeneous brain tissue density without visible abnormalities (Figure 1a). Moderate damage shows subtle changes in tissue density, which may include small hyperechoic or hypoechoic zones (Figure 1b). Intensive damage shows significant changes in density, indicating more severe hypoxic-ischemic injuries, including large hyperechoic or hypoechoic areas (Figure 1c).

Ultrasound images of the newborns’ brains were collected at the University Clinical Center Kragujevac to ensure consistency and accuracy of the results. The volumetric ultrasound recordings were converted into .jpg format with a resolution of 1024 × 1024 pixels. The used images were two-dimensional brain images in two parallel planes, which allowed for visual identification of the choroid plexus and brain parenchyma, whose densities were compared. For each patient, images were collected at 12 different time points, with key collections occurring on the first day of life within the first 6 h after birth, on the 7th day of life, and on the 100th day of life. All images were reviewed and verified by a specialist physician who performed the ultrasound examination to ensure the quality of the images.

**Figure 2:**
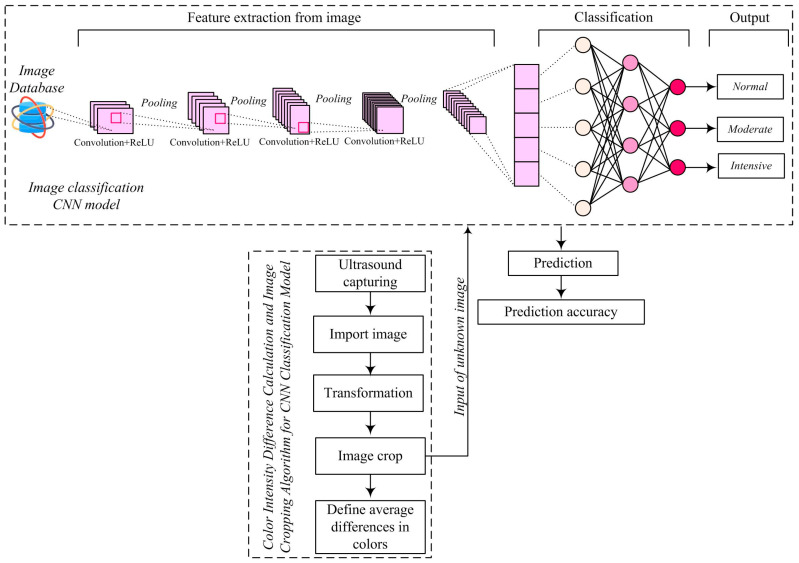
Algorithm for image classification and density determination.

The US image processing was carried out at the Center for Integrated Product and Process Development and Intelligent Systems at the Faculty of Engineering, University of Kragujevac, using MATLAB programming environment (www.mathworks.com, accessed on 20 March 2024). The applied model for classification and density difference determination on ultrasound images is shown in Figure 2. The presented model consists of two key components: a classification algorithm and a density difference determination algorithm.

The use of both approaches contributes to a more comprehensive analysis by providing unique information about the characteristics of the images. The classification algorithm focuses on categorizing images into specific groups based on their features, while the density difference determination algorithm provides quantitative information about color changes between images. The integration of these approaches allows for a better understanding and interpretation of the images, resulting in more accurate and reliable results in the classification of hypoxic-ischemic brain injuries.

#### 2.3.1. Density Difference Determination

**Figure 3:**
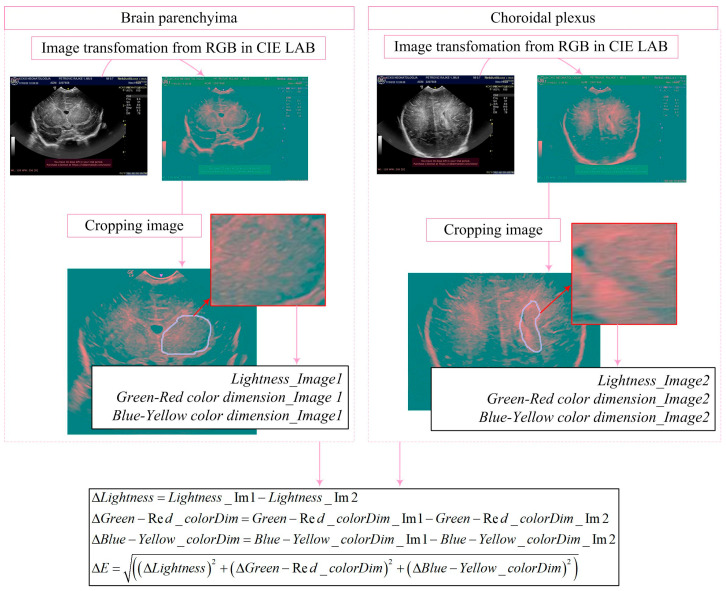
Algorithm for determining density differences between two US images.

After converting the ultrasound volumetric images to .jpg format, the images are imported into the algorithm where they are transformed from the RGB color space to the CIE Lab color space. This transformation is crucial for more accurate color analysis compared with the RGB color space, especially when dealing with the shades and intensities characteristic of medical brain images (Figure 3).

There are several reasons for choosing this method of density measurement, with some of the most important being independence from lighting, greater sensitivity to light, and more precise analysis of color shades.

The user manually defines the area of the image to be analyzed by setting the boundaries of that area. This step allows for a precise definition of the region of interest for analysis [20]:

(1) $$I_{cropped}=I\left(row_{1}:row_{2},col_{1}:col_{2}\right),$$

where

*I*—the original image in matrix format;
*row*_1_—the starting row of the region to be cropped from the original image;
*row*_2_—the ending row of the region to be cropped from the original image;
*col*_1_—the starting column of the region to be cropped from the original image;
*col*_2_—the ending column of the region to be cropped from the original image.

After the regions are selected, the mean values of the lightness, red-green, and blue-yellow channels are calculated for both cropped images. These values are crucial for further analysis of the color density differences between regions in the images and can provide important information about potential changes. Mean values are computed by summing the respective channel values for all pixels in the region and then dividing by the total number of pixels:

(2) $$\bar{L}=\frac{1}{N}\sum_{i=1}^{N}L_{i},\;\bar{a}=\frac{1}{N}\sum_{i=1}^{N}a_{i},\;\bar{b}=\frac{1}{N}\sum_{i=1}^{N}b_{i}.$$

where

$\bar{L}$—the mean value of the lightness channel;
$\bar{a}$—the mean value of the red-green channel;
$\bar{b}$—the mean value of the blue-yellow channel;
N—the total number of pixels in the selected region;
$L_{i}$—the lightness value of the *i*-th pixel in the region;
$a_{i}$—the red-green value of the *i*-th pixel in the region;
$b_{i}$—the blue-yellow value of the *i*-th pixel in the region.

After the mean values of the $\bar{L}$, $\bar{a}$, and $\bar{b}$ channels are calculated for both cropped images, the next step is to apply the Delta E CIE76 metric to quantify the color difference between them. This metric, part of the Commission Internationale de l’Eclairage (CIE) standards, measures the Euclidean distance between two color points in the CIE Lab color space. Mathematically, the color difference Δ*E* is calculated as follows:

(3) $$\Delta E=\sqrt{{\left(\Delta L\right)}^{2}+{\left(\Delta a\right)}^{2}+{\left(\Delta b\right)}^{2}},$$

where

∆*L*—the difference in the mean values of the lightness channel between the two images;
∆*a*—the difference in the mean values of the red-green channel between the two images;
∆*b*—the difference in the mean values of the blue-yellow channel between the two images.

This metric enables precise measurement of color differences and quantification of even subtle changes, which is crucial in the analysis of medical brain images.

Finally, after calculating the color difference, the images are categorized into the appropriate groups: Normal, Moderate, or intesive hypoxic-ischemic brain injury, by setting thresholds based on the measured difference. This categorization is performed using the following mathematical expression:

(4) $$Category=\left\{\begin{array}{c} Normal,\;if\;\Delta E>T_{N},\\ Moderate,\;if\;\Delta E>T_{I}\;|\wedge \Delta E\leq T_{M}\\ Intensive,\;if\Delta E\geq T_{I} \end{array}\right.,$$

where

∆*E*—the calculated color difference between the two images;
*T_N_*—the threshold for the Normal category;
*T_M_*—the threshold for the Moderate category;
*T_I_*—the threshold for the Intensive category.

After examining the existing image database and conducting the initial analysis of color density, we observed that the Δ*E* difference values were less than 10 for images categorized as Intensive, between 10 and 40 for images categorized as Moderate, and greater than 40 for images categorized as Normal. Based on this examination, we established these thresholds.

After analyzing the color difference between the cropped images, the two images are combined vertically, side by side, to form a single composite image. This combined image is then fed into a CNN for classification.

#### 2.3.2. US Image Classification Using CNN

The classification model is based on the application of a CNN and consists of two main components: feature extraction and classification (Figure 2).

**Figure 4:**
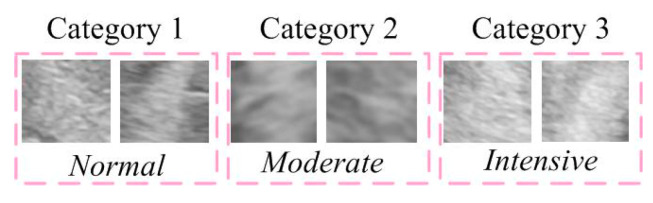
Categories in the training dataset.

For training the model, a dataset of 200 images with dimensions of 100 × 200 pixels was used. These images were categorized into three groups: Normal, Moderate, and Intensive damage (Figure 4). The collected images were organized into a unified database and classified based on their density assessment. To adequately train the network, the image database was divided into three sets: a training set (70%), a validation set (15%), and a test set (15%). In addition to the standard data split, to further assess the generalization and stability of the model, we also applied a 5-fold cross-validation.

To adequately prepare the network for introducing new examples, all images were previously scaled to the same dimensions. For each image in the dataset, with dimensions *MxNxC* where *M* and *N* are the width and height of the image, and *C* is the number of channels (e.g., 3 for RGB images), the transformation to new dimensions *PxQ* (where *P* and *Q* are the new width and height) was performed linearly, as follows:

(5) $$d_{i}\left(resized\right)=transformation\left(d_{i},\;P,\;Q\right)$$

Once the images are prepared in this manner, a CNN is constructed, consisting of layers with different structures and parameters [21]:

(6) $$L=\left(L_{1},\;L_{2},L_{3},\ldots ,\;L_{N}\right)$$

where

*L*—the entire set of layers in the CNN;
*L*_1_, *L*_2_, *L*_3_, *…*, *L_N_*—individual layers in the CNN (input layer, convolutional layers, pooling layers, and fully connected layers), each with specific roles and parameters.

Together, they enable the extraction of relevant features from the images, dimensionality reduction, and the final classification of images into appropriate categories. Layers and the characteristics of the proposed CNN model are listed in Table 1.

The convolution operation is applied to each part of the image *x* using the filter *h*, yielding the result *y*, defined as follows [15,21]:

(7) $$y\left[i,j\right]=\sum_{m}\sum_{n}x\left[m,n\right]\cdot h\left[i-m,j-n\right],$$

where

$y\left[i,j\right]$—the result of the convolution operation at position (*i*, *j*);
$x\left[m,n\right]$—the value of the image *x* at position (*m*, *n*);
$h\left[i-m,j-n\right]$—the value of the filter *h* at position (*i* − *m*, *j* − *n*).

Convolution is a key operation in CNNs that enables the extraction of features from input data. Filters, also known as kernels, are small matrices of predefined values that move across the input image. At each position, element-wise multiplication is performed between the filter values and the corresponding values in the section of the input image. The results of these multiplications are then summed to produce a single value that becomes part of the output image. This process allows the network to identify and retain relevant features such as edges, textures, and shapes. These features are crucial for tasks such as object recognition and image classification.

Pooling layers, also known as aggregation layers, typically function by selecting the maximum values within each sub-matrix of a defined size. These layers play a crucial role in CNNs by reducing the dimensionality of the output features and summarizing the information. The most commonly used method is max pooling, which extracts the maximum value within each 2 × 2 sub-matrix of the input data. The formula for max pooling is as follows [15,21]:

(8) $$y\left[i,j\right]=max\left(x\left[2i,2j\right],x\left[2i,2j+1\right],x\left[2i+1,2j+1\right]\right),$$

where

$y\left[i,j\right]$—the output value at position (*i*, *j*) after pooling;
$x\left[2i,2j\right],x\left[2i,2j+1\right],x\left[2i+1,2j+1\right]$—the values in the 2 × 2 sub-matrix of the input data.

This process not only lowers computational cost and model complexity but also helps prevent overfitting by aggregating information, making the model more resilient to variations and noise in the input data.

The ReLU (Rectified Linear Unit) function is used to introduce non-linearity into neural networks. It is defined as follows [15,21]:

(9) $$f\left(x\right)=\max\left(0,x\right),$$

where

*f(x)*—the output of the ReLU function;
*x*—the input to the ReLU function.

The softmax function is often used as the final layer in classification models to calculate the probabilities of belonging to different classes. For an input vector with *K* elements, the softmax function is defined as follows [21]:

(10) $$softmax{\left(z\right)}_{j}=\frac{e^{z_{j}}}{\sum_{k=1}^{K}e^{z_{k}}},$$

where

*softmax(z)_j_*—the output probability for the *j*-th class;
*z_j_*—the input value for the *j*-th class;
*K*—the total number of classes.

**Figure 5:**
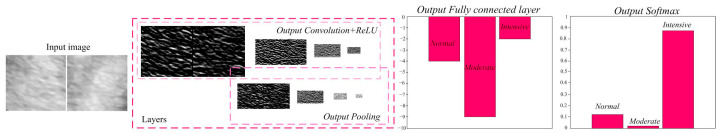
Illustration of image passing through different layers of a CNN.

An illustration of the image processing through different layers of a CNN is shown in Figure 5. The input image fed into the network passes through a series of convolutional and pooling layers, where various features of the image (such as edges, textures, and colors) are highlighted. After passing through the pooling layers, the features are summarized, and the dimensionality of the image is reduced. The output from the fully connected layer combines features from the previous layers. In this layer, all the features extracted from the preceding layers are integrated to form the final output vector. The values of this output vector are displayed in a graph and represent the network’s results before the final classification. The output from the softmax layer provides the prediction probabilities for different classes. The graph displays the probabilities for three classes, clearly showing the dominant class with a probability close to 1, corresponding to the Intensive category.

With the prepared training set, defined CNN architecture, and training options, the network training is performed. Training the network is mathematically described as the process of adjusting the weights of the neural network to minimize the loss function. This process is usually implemented using an optimization algorithm; in our case, RMSProp was used, which adjusts the weights *W* using the learning rate *η* and the gradient of the loss function *J*(*Wt*) [22]:

(11) $$W_{t+1}=W_{t}-\eta \cdot \nabla J\left(W_{t}\right),$$

where

*W_t_*—the weights of the network at the current iteration;
*η*—the learning rate;
*J*(*W_t_*)—the loss function;
∇*J*(*W_t_)*—the gradient of the loss function.

This process is repeated through multiple iterations (epochs) until the loss function is sufficiently minimized. At the end of this process, we obtain a trained network model with optimal weights tailored to solve a specific task.

**Figure 6:**
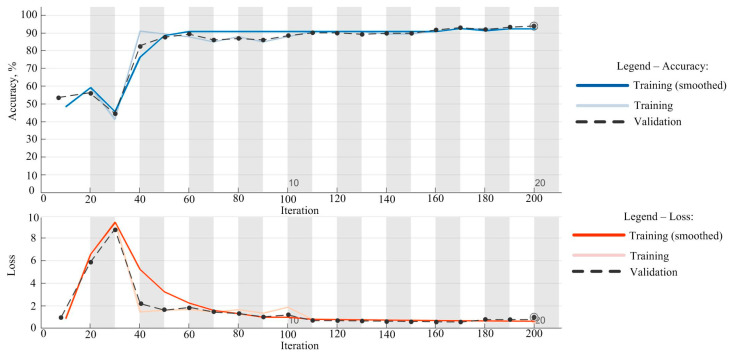
Training process.

Figure 6 illustrates the network training process. The upper graph shows the change in accuracy over 200 iterations. At the start of training, accuracy was relatively low, with a rapid increase during the first 40 iterations, reaching values close to 60%. As the number of iterations increases, training accuracy continues to rise and stabilizes above 90%. The accuracy of the validation data set follows a similar trend, with an initial surge and subsequent stabilization above 90%. The graph indicates that after approximately 40 iterations, the accuracy becomes stable and maintains a nearly constant value.

In Figure 6, the lower graph depicts the change in loss over 200 iterations. At the beginning of training, the loss was high, peaking around 20–30 iterations, after which it significantly decreased and stabilized near zero after approximately 100 iterations. The loss on the validation data set also shows a similar trend, with an initial increase, followed by a decrease and stabilization near zero. These graphs clearly demonstrate how the model’s performance improves during training, with an increase in accuracy and a decrease in loss.

The initial increase in loss during the first 30 epochs can be attributed to the process of optimization and adjustment of the model’s parameters. During early epochs, the model undergoes random initialization of weights, rapid adjustments due to a higher learning rate, and encounters complex patterns in the data. As training progresses, the model learns more efficiently, resulting in a subsequent decrease in loss and stabilization.

Based on these observations, training was stopped at 200 epochs as the model had reached stable accuracy and minimal loss, minimizing the risk of overfitting and ensuring good generalization on the validation set. Continuing training beyond 200 epochs would likely not yield significant performance improvements and could lead to overfitting.

### 2.4. Performance Evaluation

To evaluate the performance of the proposed classification model using the CNN algorithm, we analyzed a range of key indicators, including accuracy, confusion matrix, precision, recall (sensitivity), F1 score, and AUC (area under the curve) analysis [15,16,23]. The calculation of these performance indicators was based on the elements of the confusion matrix, namely True Positive (*TP*), True Negative (*TN*), False Positive (*FP*), and False Negative (*FN*).

*Sensitivity* measures how well the model detects actual positive cases:

(12) $$Sensitivity=\frac{TP}{\left(TP+FN\right)}$$

*Specificity* provides information about the model’s ability to accurately identify negative cases:

(13) $$Specificity=\frac{TN}{\left(TN+FP\right)}$$

*Accuracy* indicates the overall correctness of the model in classification, including True Positive and True Negative identifications:

(14) $$Accuracy=\frac{\left(TN+TP\right)}{\left(TP+TP+FP+FN\right)}$$

*Precision* assesses how accurate the positive predictions are as follows:

(15) $$Precision=\frac{TP}{\left(TP+FP\right)}$$

*F-measure* is calculated as the harmonic mean of precision and sensitivity:

(16) $$F-measure=\frac{2\cdot Precision\cdot Sensitivity}{Precision+Sensitivity}$$

AUC measures the model’s ability to distinguish between the presence and absence of defects, providing a value between 0 and 1, with higher values indicating better performance. These metrics together provide a comprehensive analysis, allowing us to gain a clear insight into the algorithm’s effectiveness

## 3. Results

For a clearer understanding of the data distribution monitored in this study, Table 2 presents the key values of the clinical parameters observed.

The subjects are relatively uniform in age, as indicated by the mean gestational age of 32.36 weeks, with a range from a minimum of 27 weeks to a maximum of 36 weeks. The birth weight of the neonates varies from 960 g to 3100 g, averaging 1879.10 ± 505.38 g and demonstrating considerable diversity in body mass. The Apgar score reflects the varied clinical conditions at birth, ranging from 2 to 9, with an average score of 6.79 ± 1.55. In the first 6 h of life, the average pH value is 7.23 ± 0.07, which increases to 7.38 ± 0.06 by the third week, suggesting an improvement in acid-base balance. The pCO_2_ averages 7.65 ± 1.54 mmHg in the first 6 h of life and decreases to 5.09 ± 1.01 mmHg by the third week, indicating improved ventilation. The average pO_2_ is 6.43 ± 1.84 mmHg in the first 6 h of life, rising to 7.03 ± 1.22 mmHg in the third week, indicating better oxygenation. The mean HCO_3_ values in the first 6 h of life are 20.62 ± 2.58 mmol/L, increasing to 23.88 ± 2.32 mmol/L in the third week, reflecting an improvement in metabolic balance. The BE averages −4.54 ± 6.79 mmol/L in the first 6 h of life and improves to −0.76 ± 3.41 mmol/L in the third week, showing a significant reduction in metabolic acidosis. Data distribution varies, with skewness ranging from −3.97 to 1.49, indicating differing distribution characteristics among the parameters.

Basic statistics related to the pairs, as well as the correlation, are presented in Table 3. A paired samples *t*-test analysis was applied.

In the first 6 h of life, the average pH value is 7.23 ± 0.07, increasing to 7.38 ± 0.06 by the third week, implying an improvement in acid-base balance. The pCO_2_ decreases from 7.65 ± 1.54 kPa to 5.09 ± 1.01 kPa, indicating better ventilation. Similarly, the pO_2_ rises from 6.43 ± 1.85 kPa to 7.03 ± 1.22 kPa, reflecting enhanced oxygenation. The correlation between measurements in the first 6 h of life and in the third week of life is not statistically significant for most parameters, suggesting that the changes are not consistent among the subjects. The mean HCO_3_ values increase from 20.62 ± 2.58 mmol/L to 23.88 ± 2.32 mmol/L in the third week, indicating an improvement in metabolic balance. The correlation of HCO_3_ between the first 6 h of life and the third week is close to statistical significance, suggesting a potential connection between these measurements. BE shows a significant reduction in metabolic acidosis, from −4.54 ± 6.79 mmol/L in the first 6 h of life to −0.76 ± 3.41 mmol/L in the third week. The correlation of BE between the first 6 h of life and the third week also indicates a possible association between these values, although it is not statistically significant.

Table 4 presents the differences between paired parameters, including the assessment of the mean differences, standard deviation, standard error, 95% confidence interval, *t*-value, degrees of freedom, and *p*-value (two-tailed).

For the pH parameter, a significant mean difference of −0.15 ± 0.09 was observed between values in the first 6 h and the third week of life, within a narrow 95% confidence interval. The pCO_2_ shows a mean difference of 2.56 ± 1.98, also with a narrow confidence interval. For the pO_2_ parameter, a difference of −0.61 ± 2.06 was recorded, accompanied by a wider confidence interval. The mean HCO_3_ value displays a difference of −3.27 ± 3.03, also with a wider confidence interval. BE exhibits a difference of −3.78 ± 6.79, again with a wide confidence interval. Parameters pH, pCO_2_, HCO_3_, and BE demonstrate statistically significant changes, given that the *p*-values are less than 0.05. The pO_2_ parameter has a significance value of 0.04, indicating a statistically significant change. These changes collectively suggest improvements in ventilation, oxygenation, and metabolic balance in patients in the third week of life.

Determining density differences between two ultrasound images and classification using a CNN is thoroughly described in Section 2, specifically in Section 2.3.1 and Section 2.3.2 Figure 2, Figure 3 and Figure 5 visually illustrate the processing stages, providing a visual insight into the operation of the models used.

For the purpose of evaluating the results, a confusion matrix was created. It shows that the model has a high degree of accuracy in classifying each of the three groups. The total number of correct classifications in each class is significantly higher than the number of incorrect classifications, confirming the reliability of the model (Table 5).

**Figure 7:**
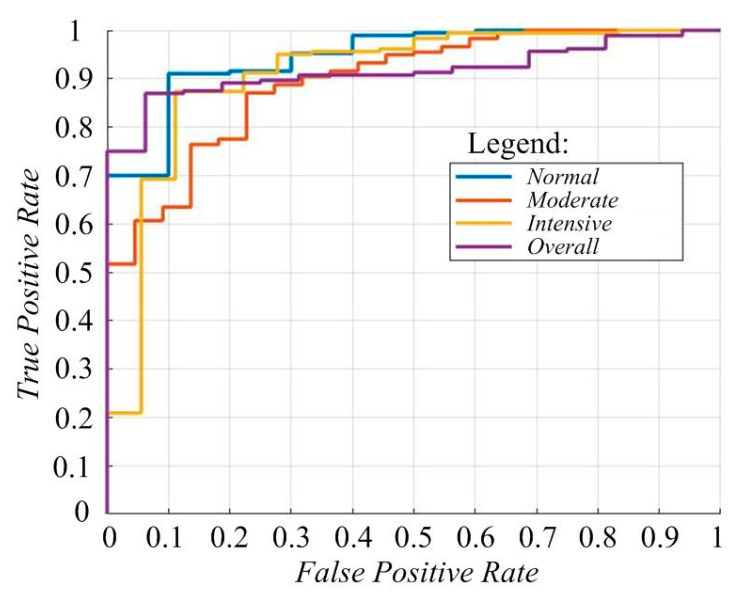
ROC curve.

The classification results of images into three groups (Normal, Moderate, and Intensive) showcase the high efficiency of the model. Specifically, the specificity for the Normal, Moderate, and Intensive groups is 96.25%, 93.75%, and 91.25%, respectively, highlighting its accuracy in identifying negative instances. Sensitivity rates are 92.5% for Normal, 87.5% for Moderate, and 87.5% for Intensive, demonstrating the model’s effectiveness in detecting positive cases. Overall accuracy is 88.56%, reflecting a balance between precision and recall. Precision values are 90% for Normal, 85% for Moderate, and 88% for Intensive, while the overall F-measure is 88.40%, indicating a successful blend of accuracy and recall in classification tasks. The AUC values stand at 0.95 for Normal, 0.89 for Moderate, and 0.91 for Intensive, with an overall score of 0.92, underscoring the model’s high performance, especially in scenarios with imbalanced classes (Figure 7).

Additionally, a 5-fold cross-validation was performed to further validate the model’s performance and generalization capability. The results of the cross-validation are summarized in Table 6.

The cross-validation results demonstrate the model’s strong performance across different classes. For Normal cases, the average accuracy is 85 ± 0.98%, with a precision of 87.29 ± 0.92%, recall of 87.27 ± 0.87%, and F-measure of 87.08 ± 0.88%. For Moderate cases, the model shows an average accuracy of 83.5 ± 1.12%, precision of 83.06 ± 1.14%, recall of 81.07 ± 1.23%, and F-measure of 81.47 ± 1.19%. In Intensive cases, the model achieves an average accuracy of 84 ± 1.05%, alongside a precision of 85.5 ± 1.07%, recall of 86.5 ± 1.10%, and F-measure of 86 ± 1.06%. The overall AUC value is 0.84 ± 0.02, with specific values of 0.85 ± 0.02 for Normal, 0.82 ± 0.03 for Moderate, and 0.84 ± 0.02 for Intensive, indicating the model’s ability to effectively distinguish between the three classes.

These results confirm the robustness and reliability of the algorithm in image classification.

## 4. Discussion

The presented study examined HIE, which represents brain damage resulting from perinatal asphyxia. The causes of perinatal asphyxia can be categorized into three main groups: prepartum, intrapartum, and postpartum. Prepartum and intrapartum asphyxia occur due to issues in gas exchange during pregnancy and labor. These conditions may be caused by pathological events related to the mother, fetus, or uteroplacental circulation. On the other hand, postpartum asphyxia occurs after birth, often due to respiratory problems, meconium aspiration, or cardiac issues in the newborn. Asphyxia is a critical factor in the development of brain ischemia and hypoxia, where insufficient blood flow, along with a lack of oxygen, triggers a series of cascading reactions. This includes the onset of acidosis, the release of inflammatory mediators, and the formation of free radicals, all of which further damage the brain [24].

The results show that the neonates included in this study initially had poor blood gas analysis findings, with low partial pressure of oxygen (pO_2_) and high partial pressure of carbon dioxide (pCO_2_) in the first 6 h of life. These findings indicate the presence of hypoxia and possible acidosis, consistent with the diagnosis of HIE. Comparing our results, particularly with data from a large study from the United States, we observe significant similarities in the clinical indicators of HIE. For example, pH values from umbilical cord blood and base deficit were within a similar range, confirming consistency in the diagnostic criteria for HIE. Blood gas parameters from umbilical cord blood, combined with brain ultrasound findings, are useful in diagnosing and assessing the severity of HIE [25].

A study conducted in Italy presents interesting findings on therapeutic hypothermia applied to neonates, with a particular focus on a group of patients who were on mechanical ventilation compared to those who were breathing spontaneously. This research showed that the base excess values, which are negative, were more pronounced in patients on mechanical ventilation [26]. A similar phenomenon was observed in our study, where the base excess values were also more negative initially. This trend can be explained by the fact that the neonates on mechanical ventilation had poorer respiratory capacity compared to those who were breathing spontaneously.

Z. Haider et al. conducted a study aiming to determine the relationship between severe umbilical artery metabolic acidosis and neonatal encephalopathy. Comparing the acid-base status parameters in HIE cases, our results show significantly better metabolic stability in our patients compared with those in their study. Higher pH and HCO_3_ values, as well as less negative BE values, suggest more effective intervention or less severe cases of HIE in our neonatal population, which may contribute to better outcomes [27].

In addition to blood gas analyses, diagnostic procedures that enable precise visualization and analysis of the brain play a crucial role in diagnosing HIE. In our study, we used cerebral ultrasonography, which has proven to be a very reliable, non-invasive, and widely accessible method. The positive effects of ultrasound application were also demonstrated by K.V. Annink et al. and M.M. Hossain et al. in their studies [28,29]. However, one of the main drawbacks of these studies is the subjectivity in assessing potential diseases, as the interpretation of ultrasound images can vary among different specialists, affecting the consistency and accuracy of diagnoses. To reduce the possibility of errors, we developed a method for assessing potential HIE through the analysis of density differences between two brain segments (brain parenchyma and choroid plexus), followed by further classification using CNN.

The significance of our research lies in the two-stage image classification process. For instance, if the CNN classifies the damage as Moderate, but the color density analysis indicates a higher value within this category, it suggests that the patient is likely to experience a faster recovery. This dual approach not only enhances the accuracy of the diagnosis but also provides a more nuanced understanding of the patient’s condition, allowing for more tailored and effective treatment plans. By integrating both CNN classification and color density analysis, we can better predict patient outcomes and improve overall care for those affected by HIE.

Density analysis in images has found applications in many fields [30]. To more accurately analyze colors compared to the RGB color space, we converted the images to the CIE Lab color space. This space allows for independent measurement and analysis of colors, resulting in more precise and objective results, independent of lighting and other external factors. Since we were comparing two cropped images, the CIE Lab space was much more suitable as it separates the luminance component from the color components, allowing for more consistent and accurate comparisons. Through multiple iterations, we determined threshold values for the density differences, based on which we could classify whether the value belongs to Normal, Moderate, or Intensive HIE [31].

After analyzing the color density difference, the images are sent to the CNN for further classification. This strategy is employed because the combination of information from the two images allows for a deeper analysis and better understanding of structural changes in the brains of neonates in HIE cases. Such an approach enables more precise and reliable diagnostic decisions based on complex criteria.

In recent years, the use of deep learning, particularly CNNs, has become increasingly significant in medical practice. CNNs are crucial in medicine because they can identify complex patterns in images and data, significantly improving the accuracy and speed of diagnosis. Their ability to automate and enhance the analysis of medical images contributes to the identification of subtle changes that might go unnoticed with traditional methods, thus enabling timely and effective patient treatment [12,13,14,15,16,32].

In many studies, CNNs have been applied, particularly in the field of neonatology. For example, K. Cui et al. developed a multimodal model based on artificial intelligence to assist clinicians in the early diagnosis of necrotizing enterocolitis. This model was trained and validated with a high accuracy of 0.94 and an area under the curve of 0.91 [33]. S. Zhao et al. developed a model for the automatic assessment of neonatal endotracheal intubation using a dilated CNN, achieving an average classification accuracy of 92.2% [34]. J.M. Brown et al. investigated whether a deep learning-based algorithm could diagnose plus disease in retinopathy of prematurity, achieving an accuracy of 91% [35]. The results of these studies suggest that algorithms can objectively and efficiently diagnose certain diseases. Evaluating the performance of our model, we demonstrated high values in precision, accuracy, F-measure, and AUC, consistent with previous research. Furthermore, the 5-fold cross-validation results also confirmed the robustness and reliability of our model, with consistent performance across different data subsets.

The architecture of our network is based on a simpler model compared with the networks presented in previous studies [29,30]. Although it consists of fewer layers, we achieved stable accuracy and loss curves during the training process after a certain number of iterations, indicating a satisfactory level of learning and generalization of the model [36]. Such a simpler architecture contributes to faster execution, requires fewer resources, reduces the tendency for overfitting, facilitates interpretation, and lowers the risk of overfitting, especially in clinical settings where rapid diagnosis is crucial.

Although we achieved significant results in color density analysis and classification using CNN, it is important to highlight some limitations of this study. First, the limited sample size may restrict the generalization of our result [37,38]. While we achieved high performance in our sample, further research is needed on a larger number of patients to better understand the general applicability of our approach. There is also a need for additional protocols to standardize or assess the reliability of result interpretation [39]. Despite our efforts to minimize these limitations, their impact should be considered when interpreting the results. Furthermore, this study included only newborns who were not delivered by emergency cesarean section to ensure a more homogeneous group, and we did not have access to data on fetal distress during labor, as these details are typically recorded in gynecological records and observed via cardiotocography (CTG).

## 5. Conclusions

This study investigated hypoxia caused by perinatal asphyxia and its impact on newborns, with a particular focus on HIE. By analyzing various clinical indicators, including blood gas analysis, pH values, and metabolic status, characteristic patterns in newborns with HIE were identified. The application of ultrasound diagnostics and the development of methods such as density difference analysis and CNN classification contributed to better diagnostics and understanding of structural changes in the brains of newborns.

Opting for simpler deep learning architectures enabled faster execution, lower resource requirements, easier interpretation, and reduced overfitting, facilitating clinical application. The results showed high algorithm performance in precision, accuracy, F-measure, and AUC, confirming the utility of simple algorithms.

Future research will focus on expanding the sample size to validate the generalizability of our findings. Successful implementation of our method in clinical practice will require collaboration with healthcare professionals, adequate training on the technology, and necessary infrastructure adjustments. These steps aim to enhance the diagnosis and treatment of HIE, providing more precise, faster, and reliable results to improve neonatal healthcare.

## Figures and Tables

**Table 1:** Layers of the proposed CNN model and their characteristics.

Layer	Stride	Output Dimensions	Output Channels	Kernel Size
Input	-	100 × 100 × 3	-	
Conv1	1	100 × 100	16	3 × 3
BatchNorm1	-	100 × 100	16	-
ReLU1	-	100 × 100	16	-
MaxPool1	2	50 × 50	16	2 × 2
Conv2	1	50 × 50	32	3 × 3
BatchNorm2	-	50 × 50	32	-
ReLU2	-	50 × 50	32	-
MaxPool2	2	25 × 25	32	2 × 2
Conv3	1	25 × 25	64	3 × 3
BatchNorm3	-	25 × 25	64	-
ReLU3	-	25 × 25	64	-
MaxPool3	2	12 × 12	64	2 × 2
Conv4	1	12 × 12	128	-
BatchNorm4	-	12 × 12	128	-
ReLU4	-	12 × 12	128	-
Fully Connected	-	1 × 1	3	-
Softmax	-	1 × 1	3	-
Classification	-	1 × 1	3	-

**Table 2:** Clinical parameters in neonates with asphyxia.

Parameter	Range	Min	Max	Mean ± S.D.	Skewness ± S.D.
Gestational week	9	27	36	32.36 ± 2.29	−0.49 ± 0.34
Weight, g	2140	960	3100	1879.10 ± 505.38	0.28 ± 0.34
Apgar score, %	7	2	9	6.79 ± 1.55	−0.93 ± 0.34
Before_pH, /	0.34	7.01	7.35	7.23 ± 0.07	−0.98 ± 0.34
After_pH, /	0.30	7.20	7.50	7.38 ± 0.06	−0.34 ± 0.34
Before_pCO_2_, kPa	9.60	3.50	13.10	7.65 ± 1.54	0.79 ± 0.34
After_pCO_2_, kPa	4.90	3.80	8.70	5.09 ± 1.01	1.49 ± 0.34
Before_pO_2_, kPa	9.10	3.10	12.20	6.43 ± 1.84	0–57 ± 0.34
After_pO_2_, kPa	7.50	3.20	10.70	7.03 ± 1.22	0.21 ± 0.34
Before_HCO_3_, mmol/L	13.40	13.20	26.60	20.62 ± 2.58	−0.61 ± 0.34
After_HCO_3_, mmol/L	10.30	19.70	30.00	23.88 ± 2.32	0.34 ± 0.34
Before_BE, mmol/L	53.30	−44.00	9.30	−4.54 ± 6.79	−3.97 ± 0.34
After_BE, mmol/L	13.50	−6.70	6.80	−0.76 ± 3.41	0.37 ± 0.34

Legend: pCO_2_—partial pressure of carbon dioxide; pO_2_—partial pressure of oxygen; HCO_3_—bicarbonate concentration; BE—base excess; S.D.—standard deviation.

**Table 3:** Paired samples *t*-test statistics and correlations.

Paired Parameters		Mean ± S.D.	Correlation	Sig.
pH	Before_pH	7.23 ± 0.07	−0.04	0.81
	After_pH	7.38 ± 0.06		
pCO_2_	Before_pCO_2_	7.65 ± 1.54	−0.17	0.25
	After_pCO_2_	5.09 ± 1.01		
pO_2_	Before_pO_2_	6.43 ± 1.85	0.14	0.33
	After_pO_2_	7.03 ± 1.22		
HCO_3_	Before_HCO_3_	20.62 ± 2.58	0.24	0.09
	After_HCO_3_	23.88 ± 2.32		
BE	Before_BE	−4.54 ± 6.79	0.25	0.08
	After_BE	−0.76 ± 3.41		

Legend: pCO_2_—partial pressure of carbon dioxide; pO_2_—partial pressure of oxygen; HCO_3_—bicarbonate concentration; BE—base excess; S.D.—standard deviation; Sig.—significance.

**Table 4:** Paired differences.

Paired Parameters	Mean ± S.D.	Std. Err.	95% Conf. Int. of the Diff.		*t*	df	Sig. (2-Tailed)
			Lower	Upper			
pH	−0.15 ± 0.09	0.02	−0.18	−0.13	−11.44	50	0.00
pCO_2_	2.56 ± 1.98	1.98	2.00	3.13	9.17	50	0.00
pO_2_	−0.61 ± 2.06	2.06	−1.19	−0.02	−207	50	0.04
HCO_3_	−3.27 ± 3.03	3.03	−4.13	−2.41	−7.62	50	0.00
BE	−3.78 ± 6.79	6.79	−5.71	−1.85	−3.93	50	0.00

Legend: pCO_2_—partial pressure of carbon dioxide; pO_2_—partial pressure of oxygen; HCO_3_—bicarbonate concentration; BE—base excess; S.D.—standard deviation; Sig.—significance.

**Table 5:** Performance metrics of the proposed classification model.

	Normal	Moderate	Intensive	Specificity	Recall	Precision	Accuracy	F-Measure	AUC
Normal	92.5%	2.5%	5%	96.25%	92.5%	90%	89.17%	91.22%	0.95
Moderate	5%	87.5%	7.5%	91.25%	87.5%	85%	87.67%	86.22%	0.89
Intensive	2.5%	10%	87.5%	93.75%	87.5%	88%	88.84%	87.75%	0.91
Overall				93.08%	89.17%	87.67%	88.56%	88.4%	0.92

**Table 6:** Performance metrics of the proposed classification model (5-fold cross-validation).

	Normal	Moderate	Intensive	Overall
Accuracy	85 ± 0.98%	83.5 ± 1.12%	84 ± 1.05%	84.17 ± 1.10%
Recall	87.27 ± 0.87%	81.07 ± 1.23%	86.5 ± 1.10%	84.95 ± 1.05%
Precision	87.29 ± 0.92%	83.06 ± 1.14%	85.5 ± 1.07%	85.28 ± 1.08%
F-measure	87.08 ± 0.88%	81.47 ± 1.19%	86 ± 1.06%	84.85 ± 1.07%
AUC	0.85 ± 0.02	0.82 ± 0.03	0.84 ± 0.02	0.84 ± 0.02

## Data Availability

The datasets used and analyzed during the current study are made available from the corresponding author upon reasonable request.
